# Speckle Tracking Echocardiography: Early Predictor of Diagnosis and Prognosis in Coronary Artery Disease

**DOI:** 10.1155/2021/6685378

**Published:** 2021-02-02

**Authors:** Maria Concetta Pastore, Giulia Elena Mandoli, Francesco Contorni, Luna Cavigli, Marta Focardi, Flavio D'Ascenzi, Giuseppe Patti, Sergio Mondillo, Matteo Cameli

**Affiliations:** ^1^Department of Medical Biotechnologies, Division of Cardiology, University of Siena, Italy; ^2^University of Eastern Piedmont, Maggiore della Carità Hospital, Novara, Italy

## Abstract

Echocardiography represents a first level technique for the evaluation of coronary artery disease (CAD) which supports clinicians in the diagnostic and prognostic workup of these syndromes. However, visual estimation of wall motion abnormalities sometimes fails in detecting less clear or transient myocardial ischemia and in providing accurate differential diagnosis. Speckle tracking echocardiography (STE) is a widely available noninvasive tool that could easily and quickly provide additive information over basic echocardiography, since it is able to identify subtle myocardial damage and to localize ischemic territories in accordance to the coronary lesions, obtaining a clear visualization with a “polar map” useful for differential diagnosis and management. Therefore, it has increasingly been applied in acute and chronic coronary syndromes using rest and stress echocardiography, showing good results in terms of prediction of CAD, clinical outcome, left ventricular remodeling, presence, and quantification of new/residual ischemia. The aim of this review is to illustrate the current available evidence on STE usefulness for the assessment and follow-up of CAD, discussing the main findings on bidimensional and tridimensional strain parameters and their potential application in clinical practice.

## 1. Background

It is widely known that echocardiography is an essential supporting tool for clinicians in the evaluation of coronary artery disease (CAD). Its application could vary between acute and chronic coronary syndromes (ACS and CCS); however, it has shown not only to aid diagnosis but also to provide useful prognostic information in this clinical setting.

The gradual introduction of speckle tracking echocardiography (STE) into clinical practice and its validation for diagnosis and risk stratification in different cardiac disease [1–4] with a great feasibility [5] have allowed to appreciate its potential additive value also for patients with CAD [6].

In fact, speckle tracking analysis is capable to assess typical ischemic subendocardial damage through several parameters: longitudinal strain (LS), which is the most used STE parameter to assess the early affection of subendocardial fibers of all cardiac chambers; bull's eye representation of left ventricular global LS (LVGLS) that provides a regional evaluation of LV injury according to coronary vascularization territories and the specific analysis of endocardial wall deformation properties with the three-layer analysis [7]. These tools could be useful to promptly guide diagnosis in uncertain cases of ACS and to provide early detection of CCS. Moreover, speckle tracking analysis could be performed on stress echocardiography (SE) images to assess subtle myocardial damage in case of doubtful stress test results or to assess myocardial viability [8]. STE was also shown to be a marker of myocardial fibrosis [9]; therefore, it could represent a noninvasive marker of myocardial postischemic scar.

The present review is aimed at providing an overview of the different clinical applications of sSTE for the evaluation of CAD, highlighting benefits and challenges of its inclusion in the diagnostic and prognostic workup of ACS and CCS.

## 2. CAD Diagnosis

The latest European Society of Cardiology (ESC) guidelines for the diagnosis and management of non-ST-elevation ACS [10] (NSTE-ACS) and CCS [11] suggest the use of speckle tracking to support diagnosis in patients referred to echocardiography for clinical suspicion of ischemic disease and absence of visual wall motion abnormalities. In fact, high sensitivity and specificity (86% and 73%, respectively) were reported for cutoff values of LVGLS > −18.8% and of LV global circumferential strain (GCS) > −21.7% (87% and 76%, respectively) to detect significant coronary stenosis in patients with chest pain and inconclusive electrocardiographic (ECG) and blood test results [12], providing an additive value to the wall motion score index (WMSI).

Accordingly, a meta-analysis including 1385 patients analyzed LVGLS ability to reveal CAD, showing satisfactory results for this noninvasive marker. The mean values of LVGLS for those with and without CAD were -16.5% [95% confidence interval (CI): -15.8% to -17.3%] and -19.7% [95% CI: -18.8% and -20.7%]. Moreover, abnormal LVGLS detected moderate-to-severe CAD with a pooled 74.4% sensitivity, 72.1% specificity, 2.9 positive likelihood ratio, and 0.35 negative likelihood ratio. The area under the curve (AUC) and diagnostic odds ratio (OR) were 0.81 and 8.5, respectively [13].

**Figure 1:**
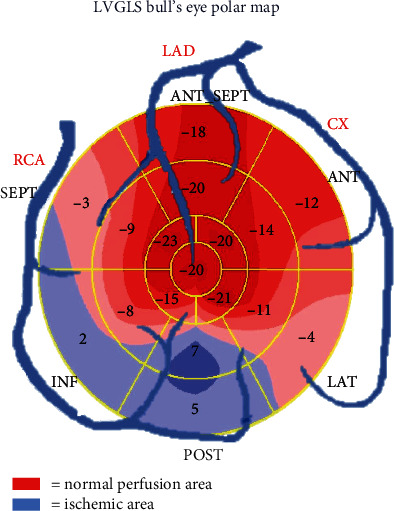
Bull's eye polar map representation of regional global longitudinal strain according to specific territories of coronary artery vascularization on a 17-segments model. The “blue” segments represent the ischemic area. ANT: anterior; SEPT: septal; ANT_SEPT: anteroseptal region; CX: circumflex coronary artery; INF: inferior; LAD: left anterior descending coronary artery; LAT: lateral; POST: posterior; RCA: right coronary artery.

What is more, LVGLS bull's eye polar maps offer an easy and quick assessment of regional distribution of myocardial necrosis through regional LS: the division in 17 wall segments from the apex to base and the visualization of a circumscribed blue area in specific segments allow to determine the distribution of blood flow-abnormalities according to the culprit coronary artery (Figure 1). Moreover, regional LS can be useful for the differential diagnosis between ACS and Takotsubo syndrome, which has typical LV strain patterns of the polar map with exclusive involvement of apical segment, and between ACS and acute myocarditis, in which polar map is quite different from that of acute myocardial infarction (AMI) since the impaired areas do not follow a typical coronary topographic localization [12].

Some authors claim that the analysis of LV regional function by segmental LS is not recommended because of less reliability and large intervendor and interobserver variabilities [14]. Therefore, it would be reasonable to use regional strain distribution to overall assess typical patterns in order to guide diagnosis, rather than evaluating the numerical segment-specific strain values, and prefer using LVGLS as the diagnostic index [1]. Moreover, high heart rate, lack of ECG tracing, and poor acoustic window (a frequent circumstance in acute settings with limited patients' movement and collaboration) strongly limit its application in the acute phase.

Therefore, in the last years, advanced imaging modalities have been proposed for the evaluation of CAD: while cardiac computed tomography (CCT) use has been recommended in the last ESC guidelines [10, 11] and National Institute for Health and Care Excellence (NICE) for younger patients with chest pain and low pretest probability of CAD, due to its greater anatomic insights and high negative predictive value (NPV) [15], cardiac magnetic resonance could be preferred for prognostic purposes in ACS and CCS [16]. In fact, in a cohort of 206 patients, the application of CCT, as first- or second-line investigation, allowed to spare 42.6% unnecessary invasive coronary angiography (ICA) and 63.7% of additional functional test (when used as first-line exam) [17]. However, CCT pitfalls still remain high costs and have low availability and a need of a specific trained team of operators and clinicians.

Of note, 103 patients with chest pain who underwent multimodality imaging evaluation with stress/rest echocardiography and CCT and LVGLS showed comparable results with CCT for the exclusion of CAD, since patients who had abnormal CCT had lower resting and peak stress GLS then those with normal CCT (14.85% ± 3.05 vs 17.99% ± 2.88, *p* ≤ 0.001; 14.89% ± 3.35 vs 18.44% ± 4.27, *p* = 0.007, respectively) [18].

### 2.1. Acute Coronary Syndromes

Being low time-consuming and easy to perform, STE could be applied also in acute settings, either before ICA, in case of uncertain diagnosis, or after revascularization for further risk stratification, if available. In patients hospitalized in a coronary care unit, the reduction of regional LV LS has shown to identify epicardial coronary lesions detected with subsequent ICA; moreover, its calculation after revascularization showed to predict the extension of myocardial necrosis due to the recent ischemia, of LV remodeling, and of postprocedural short-term and long-term complications, such as heart failure (HF) [19–22].

Noteworthy, LVGLS was demonstrated to be more accurate than WMSI in identifying NSTE-ACS patients with acute coronary occlusion who may benefit from urgent reperfusion therapy [23]. This represents an important gateway function of STE, which has been considered in the newest NSTE-ACS guidelines [10].

Particularly, LV LS polar maps are able to define the extension and localization of transmural necrosis with nonviable myocardium after AMI [24].

A study investigating the diagnostic power of LVGLS and territorial LV LS to predict CAD in patients with suspected NSTE-ACS and normal global/regional systolic function showed that GLS was significantly impaired in patients with significant coronary artery stenosis than those without (16.7 ± 3.4% vs. 22.4 ± 2.9%, *p* < 0.001) [25] and that territorial LS was able to identify the localization of coronary stenosis (left anterior descending artery (LAD), left circumflex artery (CX), and right coronary artery (RCA)); this suggests an incremental diagnostic value of GLS over the visual echocardiographic assessment of wall motion. Moreover, GLS > −19.7% showed AUC = 0.92, 81% sensitivity, and 88% specificity for detecting a significant stenosis (*p* < 0.001).

Myocardial strain by echocardiography may also facilitate the exclusion of significant coronary artery stenosis among patients presenting with suspected NSTE-ACS with unremarkable ECG findings and normal cardiac biomarkers [1]. In a study on patients referred to the emergency department with suspected NSTE-ACS, LVGLS was superior to conventional echocardiographic parameters in distinguishing patients with and without significant coronary artery stenosis (>50% luminal narrowing), with high sensitivity and NPV (AUC = 0.87, 93% sensitivity and 78% specificity, 0.74 positive predictive value (PPV), 0.92 NPV) [26]. Another research revealed that GLS and GRACE ACS risk scores were independent predictors of CAD at multivariate analysis (GLS: OR = 0.51, *p* < 0.001; GRACE score: OR = 0.93, *p* = 0.007) in patients with typical chest pain with unstable angina characteristics and a typical rise and/or fall of cardiac biomarkers, aiding in the diagnosis of NSTE-ACS [27].

LVGLS diagnostic value and capability to define myocardial infarction size were assessed in a meta-analysis including eleven studies and 765 patients, which compared LVGLS to late gadolinium enhancement (LGE) as a reference method [28]. Pooled estimates of GLS revealed a sensitivity and specificity of 77% and 86%, respectively, with an AUC = 0.70. As for the transmurality of the infarction (50% of myocardium involved was used as cutoff value), GLS showed a sensitivity and specificity of 76% and 79%, respectively, and an AUC of 0.65. These results suggest that STE could also be used as noninvasive diagnostic parameter to assess myocardial infarction area.

In addition, the analysis of LV torsion by STE has shown surprising results in patients with AMI: there is a direct correlation between torsion values and the area of the extension of myocardial infarction [29]; experimental models showed how LV torsion properties was preserved or mildly reduced for subendocardial ischemia, while being largely reduced in case of transmural ischemia. Of note, it was also considerably reduced 10 minutes after LAD occlusion (p <0.05) [30–32]. Accordingly, other authors described a clear improvement of LV torsion after percutaneous coronary intervention (PCI) [33].

#### 2.1.1. Takotsubo Syndrome

In Takotsubo syndrome (TTS) there is a transient reduction of myocardial perfusion without coronary atherosclerotic lesions, in which etiology, probably associated with emotional stress and high catecholamine and serotonin levels [34], is still a matter of research [35]; this could cause temporary LV systolic dysfunction which could mimic ACS. Typically, kinetic abnormalities are focused on the apical region (with hypo-, a-, or dyskinesia of midapical myocardial segments, sometimes associated with hypokinetic mid-segments) preserving the basal region (identifying the so-called “apical ballooning”), last few days, and then complete recovery [36]. Echocardiography has a pivotal role in identifying and in monitoring this regional kinetic impairment and overall cardiac function, in order to guide the diagnostic and therapeutic approach.

As for STE in TTS, its reduction is “circular” rather than being confined to a specific coronary region and LV twisting/untwisting properties are impaired in the acute phase [36].

It has been shown to accurately identify the recovery of myocardial dysfunction in patients with TTS 1 month after the acute phase as compared to patients with AMI [37]. However, more data are required in this field.

### 2.2. Stable CAD

To date, stable CAD was the major setting of investigation of STE among myocardial ischemic disease. Particularly, the importance of the reduction of LVGLS has been shown with rest and SE in both symptomatic and asymptomatic patients for the prediction of significant CAD [8, 38, 39].

As for rest echocardiography, GLS > −18% was prevalent in those with significant coronary lesions among 216 patients undergoing ICA for suspected CAD (*p* < 0.0001), with a 91.1% sensitivity, 63% specificity, 80.4% PPV, 81% NPV, and 80.5% accuracy for the detection of significant CAD [40]. In a similar cohort, a stratification of results for one- (AUC 0.95 for GLS> -18.44%), two- (AUC 0.9 for GLS> -17.35%), and three- (AUC 0.68 for GLS> -15.33%) vessel CAD was performed; moreover, segmental LV LS predicted the localization of the affected vessel (*p* ≤ 0.001) and had an inverse correlation with SYNTAX score that was significant for high and intermediate score (*p* ≤ 0.001) and nonsignificant for low score (*p* = 0.05) [41]. Another study of 211 subjects excluding patients with diabetes mellitus assessed the accuracy of GLS > −19% to identify coronary-specific critical stenosis [stenosis ≥ 70% in ≥1 epicardial coronary artery (≥50% in left main coronary artery)]; AUC to detect ICA stenosis was 0.818 for CX, 0.764 for LAD, and 0.723 for RCA, respectively [42].

These results confirmed the additive value of STE for the reliable detection and localization of ischemic myocardium according to coronary perfusion territories also for the study of CCS.

Radwan and Hussein showed a decrease of GLS parallel with an increasing number of coronary vessels involved in patients with stable angina and a significant positive correlation between GLS and LV ejection fraction (EF) (*r* = 0.33; *p* = 0.036); they presented a slightly inferior cutoff for GLS than other studies (GLS > −15.6% had AUC 0.88, 95% for the prediction of significant CAD; *p* ≤ 0.001), probably due to the higher cutoff of coronary stenosis considered to define significant CAD (>70% narrowing) [43].

Furthermore, two studies analyzed GLS performance in patients with normal global and/or regional wall motion on basic rest echocardiography who subsequently underwent ICA. The first study demonstrated a significant inverse correlation between GLS and SYNTAX score values (*r*^2^ = 0.38, *p* < 0.001) and identified an optimal cutoff value of GLS > −13.95% to detect high severity coronary stenosis (sensitivity = 71%, specificity = 90%, *p* < 0.001) [44]; the second one found an impaired systolic function by GLS and radial strain despite normal wall motion in patients with multivessel CAD [45].

Biering-Sørensen et al. studied 296 patients with stable angina pectoris, no previous CAD, and normal LV EF, finding that GLS was an independent predictor of CAD after multivariable adjustment for baseline data, exercise test, and conventional echocardiography (OR = 1.25, *p* = 0.016 per 1% decrease) and was able to provide an additive accuracy value to exercise test alone (AUC = 0.84 for exercise test + GLS versus 0.78 for exercise test; *p* = 0.007) [46]. Again, regional LS identified which coronary artery was stenotic, which was also confirmed in another study conducted in younger patients (mean age 51 ± 8.7 years) with suspected CAD [47].

#### 2.2.1. Three-Layer Analysis

As previously mentioned, the additional analysis of three myocardial wall layers (epicardial, midwall, and endocardial strains) by STE could be enlightening in patients with CAD, due to the peculiar distribution of ischemic damage starting form endocardium and then reaching the epicardium in the case of transmural myocardial infarction, also providing further insights for differential diagnosis (e.g., endocardial/transmural ischemia, endocardial ischemia/no ischemia, and acute myocarditis).

Therefore, several authors focused on the use of a layer-specific strain in patients with CAD, with greater utilization of circumferential and radial strain for a more reliable delineation of the layers.

Particularly, Liu et al. applied receiver operating characteristic (ROC) curves to assess the performance of three-layers STE analysis in patients with NSTE-ACS, showing that endocardial GLS and territorial LAD LS were significantly better markers (AUC = 0.91 and 0.87, respectively) of significant LAD stenosis than that in the mid-myocardial and epicardial layers in these patients [48].

Three studies also evaluated whether layer-specific circumferential strain analysis can identify scars and transmural myocardial infarction, reaching good results also after comparison with CMR [49–51].

Conversely, other authors found that epicardial and mid-myocardial LVGLS had a significantly higher diagnostic performance compared to endocardial GLS for the prediction of significant CAD (>70% coronary stenosis) in 285 patients with clinically suspected stable angina, normal EF, and no previous cardiac history [39].

Therefore, the use of three-layer analysis by STE for the assessment of coronary lesions is still controversial and its results should be taken with caution.

As an attempt to enhance diagnostic accuracy in stable CAD patients, many authors combined the use of physical/pharmacological SE and three-layer STE.

#### 2.2.2. Stress Echocardiography

The application of STE to stress echocardiography is still debated, since its feasibility could be limited by high heart rate and poor acoustic window due to patients' position; in fact, it lacks standardization and/or reference cutoffs and strongly depends on the operator's experience [1]. However, to date, there is mounting evidence supporting its use in clinical practice [3, 52].

The first studies with dobutamine SE showed that LV strain was comparable to WMSI for the diagnosis of CAD [53]. Later, LV strain showed a greater predictive value than WMSI for significant coronary artery stenoses in patients with stable CAD undergoing dobutamine SE: in one study, reduced GLS during high dobutamine dose had an AUC of 0.81 (sensitivity 89.4%, specificity 64.7%) vs. 0.78 for WMSI [54]; in another study, GLS had an AUC of 0.95 (sensitivity 94%, specificity 92%) to identify significant CAD (defined as ≥70% diameter stenosis on coronary angiography validated as hemodynamically significant by adenosine CMR) [55]. Furthermore, recovery LVGLS was the strongest predictor of obstructive CAD and was associated with positron emission tomography findings (extent, localization, and depth of myocardial ischemia) [56].

Accordingly, Park et al. found that endocardial LVGLS > −16% at recovery phase during dobutamine SE was an important predictor of significant CAD, considerably increasing sensitivity, specificity, PPV, and NPV of visual assessment alone (91%, 91%, 79%, and 96%, respectively, vs. 48%, 83%, 52%, and 81%, respectively) [57].

Nishi et al. demonstrated an association between layer-specific regional LV LS during exercise stress and functionally significant CAD as confirmed by invasive fractional flow reserve in stable patients. Moreover, the combination of endocardial LV LS and percent change in the endocardial-to-epicardial LV LS ratio at early recovery phase offered an incremental diagnostic value to visual estimation of LV wall motion for the detection of the ischemic territory (AUC = 0.75 vs. 0.61 of visual estimation alone, *p* = 0.006) [58].

In 132 patients undergoing adenosine SE and ICA, endocardial, midventricular, and epicardial LVGLS had similar diagnostic values, with high specificity, even though showing modest sensitivity, which could limit its clinical application [59].

An important use of STE during stress echocardiography in clinical practice could be the assessment of subtle myocardial injury in patients with cardiovascular risk factors [60].

Interestingly, two researches evaluated the use of STE during SE in almost-entirely women cohorts: the first one found significantly impaired values of GCS, global radial strain and strain rate, and GLS in patients with angiographically confirmed CAD and a positive exercise stress echocardiography as compared with controls, showing that a combination of GLS, GCS, and standard deviation of the longitudinal strain time-to-peak had very high accuracy for the detection of CAD (AUC = 0.96, sensitivity 97%, specificity 86%) [61]. The other study assessed whether STE during SE could help in the diagnosis of microvascular angina, showing that the most discriminative parameter for microvascular angina during SE was GCS [62].

#### 2.2.3. The Choice between Global and Regional Strain

Even though the abovementioned studies showed a valuable diagnostic power of LVGLS for the study of CAD, since a reduction of LVGLS in patients with typical angina is highly suggestive of CAD, the key for the diagnosis of stable CAD is represented by the additive value of regional strain analysis. However, it is characterized by high variability making its interpretation more challenging and requiring experience, also considering that its sensitivity could vary among different LV segments depending on their location and their echocardiographic visualization (often limited by poor acoustic window) [63]. This is why many authors chose to use more easily and rapidly performing LVGLS that we endorse in order to avoid under- or overestimation of myocardial damage; however, we recommend the integration of STE with clinical data to enhance diagnostic probability.

## 3. Prognosis

The evaluation of patients with acute and chronic CAD using STE has shown to improve the prognostic assessment of these patients, particularly those with preserved EF, as STE is able to predict cardiac dysfunction prior to EF reduction [64]. This is a crucial point, since the development of HF and cardiac death as a consequence of AMI strongly depends on the extent of myocardial damage.

First of all, STE has shown an association with after-ACS event clinical outcome in different studies: a LVGLS > −13% measured during the index hospitalization was a predictor of event-free survival in a cohort of both STE-ACS and NSTE-ACS [65], while LVGLS > −14% predicted admissions for acute HF and cardiovascular mortality in patients with AMI [66].

In 70 patients with NSTE-ACS < 72 hours, an impaired baseline LVGLS and its lack of improvement 24 hours after coronary revascularization were associated with negative LV remodeling (defined as lack of improvement of LV function, with increase in LV end-diastolic volume ≥ 15%) (OR = 4.3, *p* < 0.0001; OR = 1.45, *p* < 0.01, respectively) [21].

Moreover, in a large study of patients with recent AMI, LVGLS and strain rate were significantly and independently correlated with all-cause mortality, reinfarction, revascularization, and HF hospitalization at 3-year follow-up (OR = 4.5 for LVGLS < −15.1% and 4.4 for LV strain rate > −1.06 s^−1^), and LVGLS was superior to LV EF and WMSI after multivariate analysis [67].

Furthermore, van Mourik et al. demonstrated the additional value of STE over visual echocardiographic evaluation for the accuracy in the detection of postinfarct scars in a cohort of patients analyzed around 110 days after STE-ACS [68]. An early assessment of residual ischemic injury and myocardial viability after AMI can help to optimize the therapeutic management in order to prevent serious complications, such as LV remodeling with development or progressive worsening of HF, arrhythmias and sudden cardiac death, or to identify patients to refer for cardiac surgery, LV mechanical assistance treatment, or preventive intracardiac defibrillator implantation.

Importantly, the evaluation of transmurality of myocardial ischemia and the degree of endocardial damage play an important role in the prognosis of CAD not only in the acute phase but also during follow-up, in which STE could be of great utility for its high availability and rapidity of execution.

In fact, Joyce et al. used STE for the evaluation of 105 first STE-ACS patients treated with primary PCI at baseline and during follow-up (together with 3-month SE and 1-year ICA); they found that patients with significant angiographic CAD at 1-year had greater worsening in global LVGLS during SE from rest to peak (−16.8 ± 0.5% to −12.6 ± 0.5%) compared with patients without significant CAD (−16.6 ± 0.4% to −14.3 ± 0.3%), with an optimal cutoff of global variation ≥ 1.9% (AUC 0.70; sensitivity, 87%; specificity, 46%); higher segmental *Δ*GLS was independently associated with significant CAD (OR 1.1) [69].

Also, a prospective study comparing 94 patients with a first AMI and 137 patients with stable CAD, all of whom had undergone coronary revascularization, showed that in stable CAD patients, the addition of endocardial LVGCS > −20% to baseline characteristics and EF into a regression model significantly improved the prediction of cardiac events (AUC = 0.86, sensitivity: 79%, specificity: 84%); conversely, the same analysis in AMI patients was unsuccessful to increase the predictive power for cardiac events [70].

Notably, in a small population of after-STE-ACS, three-layer STE was applied to assess the strain gradient between the three layers as a marker of irreversible transmural damage and of myocardial viability, with ROC curves endocardial LS having an AUC = 0.69 and strain gradient having an AUC = 0.73 for myocardial viability [71].

## 4. Postsystolic Shortening

Some authors consider the calculation of postsystolic shortening (PSS) during strain analysis in patients with CAD as equally or more important to commonly used LV strain, since its presence is a characteristic feature of myocardial ischemic dysfunction [72].

PSS is defined as myocardial shortening that occurs after end-systole and is observed mainly during isovolumic relaxation [73]. This relies on the fact that regional contraction of the myocardium depends not only by inherent contractility of the concerned myocardium but also by tension from the surrounding myocardium. Therefore, in case of reduced regional contractility because of ischemia, the amplitude of shortening during ejection time decreases, and early systolic lengthening (ESL) and PSS are observed in the ischemic myocardium.

In some case of myocardial ischemia when regional wall motion abnormalities are not seen on visual assessment, the analysis of the LV strain curve show PSS, appearing as the peak of regional LS that occurs after aortic valve closure (AVC).

The mostly used parameter to quantify PSS is postsystolic index, which is calculated as follows: ([peak postsystolic strain] − [end‐systolic strain])/(peak strain or maximum strain change during the cardiac cycle), showing the ratio of the amplitude of PSS to total shortening. The time from aortic valve closure to peak postsystolic strain is used as another parameter [74].

The assessment of PSS is valuable in identifying acute ischemia, because PSS occurs in the myocardium with regional contractile dysfunction [75]. It was found to be a reliable index for the diagnosis of CAD, at rest and during SE [46], and also to be associated with prognosis in patients with stable angina [76].

## 5. Other Cardiac Chambers

Even though the most studied cardiac chamber for the evaluation of CAD is the LV, representing the largest part of myocardium and being responsible of cardiac pump function and output, the other cardiac chambers could be either directly involved in ischemic cardiac damage (particularly, left atrium (LA) in the case of CX lesions and right ventricle (RV) in the case of RCA) or secondarily affected due to postischemic acute or chronic HF [77].

As the application of STE to LA and RV has been increasingly performed for the evaluation of HF, valvular disease, hypertension, etc. [78–80] showing great feasibility regardless of the operator's experience [81], it has also recently been extended to patients with CAD.

### 5.1. Left Atrial Strain in CAD

In 68 patients with AMI treated with emergent or urgent PCI, peak atrial longitudinal strain (PALS) was lower in patients with a CX culprit lesion than those with culprit lesions in other vessels, whereas the LA volume index did not show any difference. This confirms the importance of LA strain over dimensional measures for the early diagnosis of myocardial damage [82].

In a small study involving patients with stable CAD undergoing ICA, PALS and peak atrial contraction strain (PACS) were significantly reduced in patients with SYNTAX score ≥ 33; notably, these parameters had a close negative correlation with such parameter (*r* = 0.861; *p* < 0.001) [83]. LA strain was also related to clinical outcome in a cohort of patients with AMI undergoing PCI [84].

Meanwhile, in patients with typical Takotsubo syndrome who underwent transthoracic-Doppler echocardiography during the acute phase and at follow-up (32 ± 18 days later), PALS was transiently impaired at baseline and was associated to in-hospital complications. Moreover, LA strain improved parallel to the dynamic improvement of LV GLS, following the typical feature of a transient myocardial damage of the disease [85].

### 5.2. Right Ventricular Strain in CAD

As previously mentioned, RV dysfunction was found by STE in 87 patients with CAD involving RCA, in whom free wall RV LS was an independent predictor of RCA involvement at multivariate analysis (OR = 1.07; 95%; *p* = 0.02) [86]. Therefore, it could be used as a reliable marker of RV dysfunction in patients with inferior AMI.

Moreover, RV involvement has shown significant prognostic consequences in CAD: patients with acute MI complicated by cardiogenic shock showed a worse prognosis if RV dysfunction by echocardiography was present [87]. Antoni et al. also showed that a reduction of RV strain was an independent predictor of death, reinfarction, and HF hospitalization (hazard ratio = 1.08) in patients with AMI treated with PCI; finally, RV strain provided an incremental value to clinical information, infarct characteristics, LV function, and RVFAC [88].

## 6. 3D Strain

The advances in cardiac imaging and the development of new devices have led to more availability of three-dimensional (3D) echocardiography, which provides further insights on cardiac anatomy and is considered superior to 2D echocardiography for the assessment of cardiac geometry. However, 3D strain value in clinical practice is still debatable, also due to vendor-dependency and the lack of standardization.

However, recent studies suggested a potential role of 3D strain for the evaluation of patients with stable and unstable CAD.

A recent investigation involving 255 STE-ACS patients undergoing PCI demonstrated that 3D-LVGLS was the strongest predictor of LV reverse remodeling (OR = 1.43, *p* = 0.02) and major adverse cardiac events (OR = 1.44, *p* < 0.0001), being superior to bidimensional LVGLS for the prediction of outcome [89]. Similar results on 3D strain as the index of future LV reverse remodeling were showed in another STE-ACS cohort [90].

Moreover, in patients with NSTE-ACS, 3D STE performed prior to ICA showed that 3D GLS > −13.50% could detect those with significant coronary disease (AUC = 0.84) [91].

Finally, in 130 patients with stable angina pectoris, 3D GLS was correlated with Gensini score, with 88.9% sensitivity and 92.9% specificity being observed for a GLS > −10%; while global area strain (GAS), a new feature of 3D echocardiography which integrates longitudinal and circumferential deformation, had 97.2% sensitivity and 88.1% specificity for a cutoff value > −21% to detect critical CAD (estimated as Gensini score ≥ 20) [92].

Despite these promising results, the diagnostic value of 3D GLS was lower than that of 2D GLS in a recent meta-analysis on the detection of myocardial infarction size [28]; this suggests that more consolidated researches are warranted to define the 3D usefulness in this clinical setting.

## 7. Limitations

The major limitation of STE is the lack of defined cutoff values for its confident use in different clinical settings.  Table 1 shows medium cutoff values of several strain parameters proposed in the aforementioned studies on patients with CAD; however, these values need an external validation to become generalizable; LA and RV strain cutoffs require further research to be identified. Vendor dependency could be considered partially solved after the publication of the European Association of Cardiovascular Imaging (EACVI) standardization documents for all chambers' deformation imaging [93]. Also, negative values of LV and RV strain are currently matter of discussion, since the use of negative values could result in some confusion, especially when it comes to expressing majority and minority criteria, or could expose to important mistakes during the data collection for missing minus typing. We agree with this opinion and understand the choice of some authors to report absolute values in their research papers; however, in our personal practice, we still prefer to use negative values of ventricular strain since it currently is the most standardized method based on the international committee documents. Moreover, the use of a negative sign is important to differentiate ventricular strain, which describes contractile function, being negative in order to reflect myocardial fiber shortening, from left atrial strain, which describes relaxation properties as myocardial fiber distension.

Furthermore, STE maintains the common limitations of bidimensional echocardiographic measures, such as image quality, operator dependency, and load dependency (lower than LV EF). These limitations could be overcome by the use of 3D echocardiography. However, validated data and standardization among different vendors are necessary to extend its applicability beyond research purposes.  Table 2 resumes the benefits and drawbacks of using STE for the study of CAD.

## 8. Conclusions

**Figure 2:**
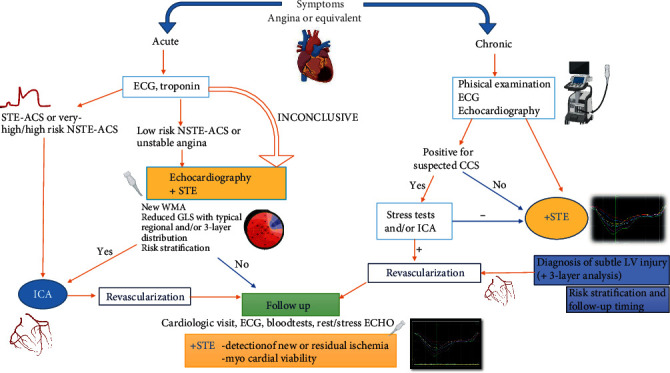
Potential integration of speckle tracking echocardiography as additive tool in diagnostic and follow-up algorithms of acute and chronic coronary syndromes. Large prospective studies are needed to validate this algorithm and investigate its impact on clinical outcome.

Beyond ECG and biomarkers, echocardiography is a milestone for the evaluation of CAD in acute and chronic settings. STE could provide an additive value over visual wall motion assessment both for diagnostic and prognostic assessment, and the inclusion of LVGLS in clinical diagnostic workup of these patients is supported by plenty of evidence and clear advantages overweighing the intrinsic limitations of STE technique (Figure 2). However, further studies are needed to confirm the potential value of other chambers' strain. Future experts' consensus to identify reference values of LV strain parameters in CAD is highly expectable for a definitive standardization of their use.

## Figures and Tables

**Table 1:** Medium cutoff values of strain parameters for diagnosis and prognostic assessment of coronary artery disease based on the available literature.

	Diagnosis		Prognosis	
	Acute	Chronic	Acute	Chronic
GLS	-17.82% [12, 13, 26, 27]	-17.41% [37, 39, 41–43] SE: -16.75% [55, 56, 58]	-13.32% [20, 21, 64–66]	—
GCS	-17.35% [12, 19]	—	-13% [19]	-20% [69]
GRS	—	—	—	—
Regional LS	—	-20.45% [44, 64]		—
Torsion	1.39 degrees/cm	—	—	—
PSS	-13.9% [73]			
PALS	—	—	—	—
fwRVLS	—	—	—	—
3D strain echocardiography	3D GLS: -11.75% [89, 90] 3D GAS: -21% [90]			

fwRVLS: free-wall RVLS; GAS: global area strain; GCS: global circumferential strain; GLS: global longitudinal strain: GRS: global radial strain; PALS: peak atrial longitudinal strain; PSS: post systolic-shortening; SE: stress echocardiography.

**Table 2:** Benefits and drawbacks of using speckle tracking echocardiography for the evaluation of coronary artery disease.

Advantages	Disadvantages
Noninvasive	Lack of standardization and defined cutoff values
Availability and repeatability	
Rapidity	Operator-dependence
Portability	Acoustic window-dependence
Low costs	Challenging in case of high heart rate and arrhythmias
Semiautomatic and angle-independent (more reliable than 2D-echo)	Lower spatial resolution than other imaging methods
Early diagnosis with regional localization of myocardial injury	
Differential diagnosis with bull eye-specific patterns	
